# A reinvestigation of the reference frame of the tilt-adaptation aftereffect

**DOI:** 10.1038/srep01152

**Published:** 2013-01-28

**Authors:** Sebastiaan Mathôt, Jan Theeuwes

**Affiliations:** 1 Aix-Marseille Université, Laboratoire de Psychologie Cognitive; 2 Le Centre National de la Recherche Scientifique; 3 VU University Amsterdam, Department of Cognitive Psychology

## Abstract

The tilt-adaptation aftereffect (TAE) is the phenomenon that prolonged perception of a tilted ‘adapter’ stimulus affects the perceived tilt of a subsequent ‘tester’ stimulus. Although it is clear that TAE is strongest when adapter and tester are presented at the same location, the reference frame of the effect is debated. Some authors have reported that TAE is spatiotopic (world centred): It occurs when adapter and tester are presented at the same display location, even when this corresponds to different retinal locations. Others have reported that TAE is exclusively retinotopic (eye centred): It occurs only when adapter and tester are presented at the same retinal location, even when this corresponds to different display locations. Because this issue is crucial for models of transsaccadic perception, we reinvestigated the reference frame of TAE. We report that TAE is exclusively retinotopic, supporting the notion that there is no transsaccadic integration of low-level visual information.

A central issue in research on visual perception and eye movements is the extent to which a detailed representation of our visual surroundings is preserved across eye movements^1^,^2^. We can split this issue into two questions. Firstly, when making eye movements, do we preserve a representation of our entire visual environment, or only of a subset of objects, presumably those that are in the focus of attention? And secondly, is this representation rich, in the sense that it contains detailed information about visual features, such as orientation, colour, form, etc.? Or is this representation sparse, perhaps barely more than some positional information, or ‘attentional pointers’^3^, to serve visually guide action^4^,^5^,^6^,^7^,^8^?

There is broad consensus on the first question: We do not maintain a cognitive representation of our entire visual environment across eye movements, at least not if we equate representation with visual awareness^9^. Rather, as experiments on change detection^10^,^11^,^12^ and inattentional blindness^13^,^14^,^15^ have shown, we are only aware of a very limited number of objects at a time. This is true for perception in general, but also applies to transsaccadic integration: Only a very limited number of objects are preserved across eye movements^16^,^17^.

However, there is considerable controversy about the nature of the representations that underlie transsaccadic integration: If we consider those few objects that, at any one time, are subject to transsaccadic integration, what properties of those objects are preserved? The majority of studies point towards the counterintuitive conclusion that very little information about objects is retained across saccades, even about those objects that are in the focus of attention^18^,^19^,^20^,^1^. This has been demonstrated particularly elegantly in a classic study by McConkie and Zola^20^, in which participants read words consisting of letters with randomly alternating case (LiKE tHis). The crucial manipulation was that letter case was reshuffled when participants made an eye movement (e.g., from LiKE tHis to LikE ThiS). The surprising finding was that participants frequently failed to notice this change. Since they were reading, we may assume that participants were paying attention to the words. Yet this seemingly obvious change went unnoticed, even though it occurred to an attended stimulus in foveal vision. This striking finding clearly suggests that there is little or no transsaccadic integration of detailed object features: We do not integrate a picture-like retinal image from one fixation to the next.

However, another line of research converges on the opposite conclusion, namely that low-level features are preserved across saccades as well, at least to some extent. The most convincing evidence for transsaccadic integration of low-level features comes from studies on adaptation aftereffects across saccades^21^,^22^,^23^,^24^,^25^,^26^,^27^. In general terms, an aftereffect is the phenomenon that, after prolonged exposure to an ‘adapter’ stimulus, people perceive a subsequently presented ‘tester’ stimulus as being ‘pushed away’ from the adapter stimulus in the adapted feature dimension. For example, in the case of faces, this means that people perceive an androgynous tester face as male, when it is preceded by a female adapter face (the face-adaptation aftereffect^28^). In the case of orientation, this means that people perceive a vertical stimulus as being tilted clockwise, when it is preceded by a counterclockwise adapter stimulus (the tilt-adaptation aftereffect^29^). The same principle holds for a wide variety of stimulus features, such as form, direction, motion, and numerosity.

It has been long recognised that adaptation aftereffects are partly location-specific, such that the effect is strongest when the adapter and tester are presented at the same location. However, it was not clear whether such effects are tied to a retinal location (retinotopic) or a location ‘out there’ in space (world centred or spatiotopic). Therefore, in an influential study, Melcher^23^ set out to investigate the extent to which aftereffects are spatiotopic. He investigated four types of adaptation aftereffects (contrast-adaptation, tilt-adaptation, form-adaptation, and face-adaptation) using a straight-forward paradigm. First, an adapter stimulus was presented at fixation. After the adapter had been extinguished, participants made a saccadic eye movement. Finally, a tester stimulus was presented. Participants identified the tester stimulus (for example by reporting gender in the case of faces), and the strength of the adaptation aftereffect (i.e. the influence of the adapter on the perception of the tester) was the dependent measure. Crucially, the tester was presented either at the same spatial location as the adapter (the spatiotopic condition), or at a control location. The striking finding was that the extent to which there was a spatiotopic aftereffect depended on the complexity of the stimulus: There was no spatiotopic adaptation for contrast, some for tilt, more for form, and almost full spatiotopic adaptation for faces. Melcher^23^ interpreted this result as showing that detailed visual features are preserved across saccades, even relatively low-level visual features, such as orientation, although to a lesser extent^30^.

Although spatiotopic adaptation was most pronounced for faces^23^, subsequent research focused primarily on the tilt-adaptation aftereffect (TAE), which has been used to investigate the mechanisms that underlie transsaccadic perception in general. For example, based in part on TAE experiments it was concluded that transsaccadic integration relies on a ‘remapping’ process that starts before the onset of a saccadic eye movement^24^, and occurs primarily for objects that are in the focus of attention^25^. In other words, the premise that spatiotopic TAE exists has guided experiments and thinking of many researchers.

Yet recently a number of authors have reported that these same aftereffects are exclusively retinotopic, without any corresponding spatiotopic component^31^,^32^,^33^,^34^,^35^,^36^. In other words, these authors suggest that most forms of adaptation (including tilt adaptation) are low-level phenomena, which occur in a retinotopic frame of reference, and are not subject to transsaccadic integration (i.e. are not remapped). Because of the impact of experiments on spatiotopic TAE, the debate over whether this effect exists at all is of considerable theoretical significance. Therefore, the aim of the present study was to reinvestigate the reference frame of TAE. In our view, there are two troubling factors in most previous research on the reference frame of adaptation aftereffects. Firstly, most paradigms did not include control locations that were matched with respect to eccentricity and position relative to saccade direction. For example, in the design used by Melcher^23^, the control location was always in the same direction as a preceding eye movement, whereas the spatiotopic location was in the opposite direction. Although it is not obvious why this should bias the results towards finding a spatiotopic effect, it is nevertheless difficult with this design to conclusively dissociate location-specific from generalised effects—Some adaptation is generally observed throughout the visual field. To address this problem we used separate, carefully matched control locations for both the spatiotopic and retinotopic locations. Secondly, the nature of adaptation experiments allows for a confounding influence of the observers' expectations: Trial progression is slow, experiments are tedious, and responses are generally not speeded. Therefore, observers have ample opportunity to contemplate the goals of the experiment and inadvertently adjust their responses accordingly. This may confound results even if observers are naïve, but is particularly problematic when many^33^ or even most^37^ of the observers are also authors. To alleviate this issue, all observers in the crucial experiments (2 and 3) were naïve and without training as psychophysical observers. Furthermore, observers were instructed to respond as quickly as possible, in order to minimize the opportunity for excogitation. In other words, relative to previous studies, we de-emphasized the subjective aspect of the task, and instead relied on accuracy and speeded response times, which are largely implicit measures.

Aside from these points, we used the same overall methodology as used in many of the studies mentioned above. First, a task-irrelevant adapter stimulus (a tilted Gabor-like stimulus), was presented for several seconds to elicit tilt adaptation. Next, observers made a saccadic eye movement, so that the (former) location of the adapter stimulus was displaced on the retina. Finally, a tester stimulus (another tilted Gabor-like stimulus) was presented at either the adapter location (a spatiotopic match), the location that retinotopically matched the adapter location (a retinotopic match), or one of two control locations. Observers reported the orientation of the tester stimulus as quickly as possible. Due to tilt adaptation, we expected in general that observers would respond less accurately and less quickly when tester and adapter were tilted in the same direction, compared to when they were tilted in opposite directions: This is the classic tilt-adaptation aftereffect (TAE). Crucially, we investigated whether TAE is most pronounced when tester and adapter were presented at spatiotopically matching locations (the same locations on the display, but different locations on the retina), retinotopically matching locations (the same locations on the retina, but different locations on the display), or whether TAE has both a retinotopic and a spatiotopic component.

In total, we conducted three experiments. Experiment 1 was a validation experiment, in which we determined experimental parameters that provided a sensitive measure of TAE. In Experiment 2, we showed that TAE is retinotopic: A strong TAE was observed when the adapter and tester were presented at the same location on the retina and different locations on the display, but not when they were presented at the same location on the display and different locations on the retina. In Experiment 3, we replicated this result, and in addition showed that the retinotopy of the TAE is persistent.

## Results

### Experiment 1: validation

The aim of Experiment 1 was to determine the combination of tester orientation, adapter orientation, and dependent measure (TAE derived from accuracy or response times) that provides the most sensitive measure of TAE. The full experimental paradigm is described under *Methods*. In brief, observers were presented successively with an adapter and a tester stimulus, while they maintained fixation at a single location. The adapter and tester could be presented either at the same location, or at different locations. This allowed us to measure the location-specific component of TAE.

Trials were excluded based on the following criteria: Gaze deviated more than 2° from the fixation dot (7.2%); Response times were below 50 ms (-) or 1500 ms (0.3%). In total, 92.6% of all trials were included in the analysis. An alpha level of .05 is used throughout the analyses.

**Figure 1:**
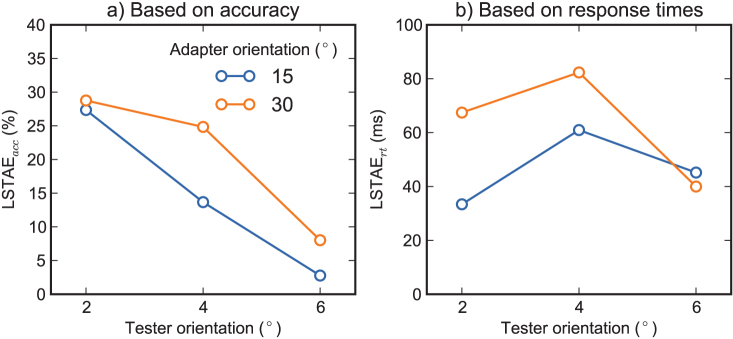
Results of Experiment 1 (validation). Quantitatively, each combination of adapter orientation, tester orientation and dependent variable yielded a tilt-adaptation aftereffect, which illustrates that the effect is substantial and highly robust. The most reliable effect was observed with the 2° tester orientation, 30° adapter orientation, and TAE derived from accuracy as dependent variable.

We quantified the location-specific component of the tilt-adaptation aftereffect (LSTAE; see *Methods*), measured using response times (LSTAE_rt_) or accuracy (LSTAE_acc_). A repeated measures Analysis of Variance (ANOVA) was conducted with tester orientation and adapter orientation as within-subject factors, and LSTAE_rt_ as dependent variable. This revealed no effects (Figure 1b), suggesting that the strength of LSTAE_rt _was not reliably dependent on the adapter and tester orientation. A similar analysis was conducted with LSTAE_acc_ as dependent variable. This revealed an effect of tester orientation, *F*(2,14) = 5.4, *p* = .0183, such that LSTAE_acc _was larger for small tester orientations. Tentatively, there was a trend towards an effect of adapter orientation, *F*(1,7) = 4.8, *p* = .0656, such that LSTAE_acc _was largest for the 30° adapter orientation (Figure 1a).

To investigate which dependent measure was most sensitive, we transformed both accuracy and response times to *Z*-scores, separately for each participant. Next, using *Z*-scores, but otherwise as described under *Methods*, we determined LSTAE per participant across all tester and adapter orientations. A two-tailed paired samples *t*-test showed that LSTAE_acc_, *M* = .49, was higher than LSTAE_rt_, *M* = .25, *t*(7) = 7.3, *p* = .0002, illustrating that TAE derived from accuracy was a more sensitive measure than TAE derived from response times.

Finally, we checked whether there was a robust location-specific TAE for the optimal combination of dependent variable (LSTAE_acc_), tester orientation (2°), and adapter orientation (30°). Assuming a uniform distribution, a lower bound of 0 (no difference), and an upper bound of 50 (maximum difference), we determined the Bayes factor: *Bf* = 74.8, *M* = 29, *SE* = 10. Following Jeffreys^38^,^39^, this constitutes “very strong evidence” for the existence of TAE (H_a_).

To summarize the results of Experiment 1, it proved easy to elicit a substantial, location-specific TAE using a speeded response time task. The effect was found, at least qualitatively, across all tester and adapter orientations, and for TAE derived from response times as well as accuracy. However, the most robust results were obtained using TAE derived from accuracy as dependent measure, a tester orientation of 2°, and an adapter orientation of 30°. We therefore used these parameters, and this dependent measure for Experiments 2 and 3.

### Experiment 2

The aim of Experiment 2 was to determine the reference frame of TAE. The full experimental paradigm is described under *Methods*. In brief, observers were presented successively with an adapter and a tester stimulus. Crucially, observers made a saccadic eye movement in between the presentation of the adapter and the tester. This allowed us to investigate whether TAE is most pronounced when adapter and tester are presented at the same location on the display (spatiotopic) or the same location on the retina (retinotopic).

Trials were excluded based on the following criteria: The eyes deviated more than 2° from the expected location during the trial (10.3%); Saccade latencies were below 50 ms (3.8%) or above 500 ms (1.0%); Response times were below 50 ms (<0.1%) or above 1500 ms (1.1%). In total, 83.7% of all trials were included in the analysis.

**Figure 2:**
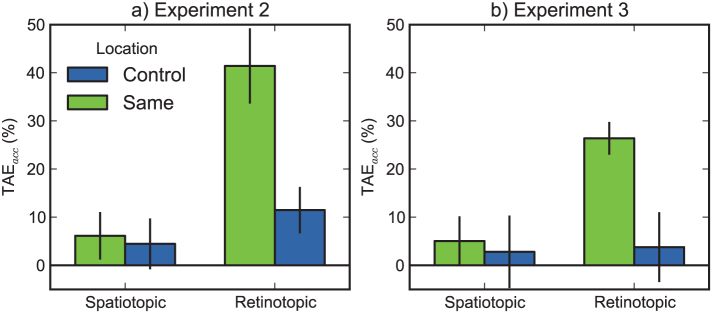
Results of Experiments 2 and 3. In both experiments, there was a significant tilt-adaptation aftereffect (the difference between ‘same’ and ‘control’) in the retinotopic condition, but not in the spatiotopic condition. Error bars reflect 95% within-subject confidence intervals^47^.

Based on the results of Experiment 1, we focused on the tilt-adaptation aftereffect (TAE; see *Methods*) as measured using accuracy (TAE_acc_), although we analysed response times (TAE_rt_) as well. A repeated measures ANOVA was conducted with condition (spatiotopic, retinotopic) and location (same, control) as within-subject factors and TAE_acc_ as dependent variable (see Figure 2a and Table 1). This revealed an effect of condition, *F*(1,7) = 35.4, *p* = .0006, such that TAE_acc_ was higher in the retinotopic than in the spatiotopic condition, and an effect of location, *F*(1,7) = 21.8, *p* = .0023, such that TAE_acc_ was higher at the same than at the control locations. Crucially, there was a condition by location interaction, *F*(1,7) = 14.6, *p* = .0066, reflecting that the main effects are driven by a difference between the actual and mirror retinotopic conditions. A similar analysis with TAE_rt_ as dependent variable, revealed only a main effect of condition, *F*(1,7) = 8.0, *p* = .0253. The results from TAE_rt _qualitatively matched those from TAE_acc_.

Because null-hypothesis testing does not, by itself, allow for claims about the absence of an effect, we verified the existence or non-existence of location-specific TAE more rigorously. We determined the Bayes factor (Bf) for the difference between same and control trials in both the retinotopic and spatiotopic condition. We assumed a uniform distribution with realistic lower and upper bounds for the expected difference. We set the lower bound to 0, since this reflects an absolute lack of location-specific TAE. We set the upper bound to 50, since this reflects the largest possible location-specific TAE. For the spatiotopic condition, this gave us the following: *M* = 1.67, *SE* = 3.99, *Bf* = 0.14. Following Jeffreys^38^,^39^, this indicates “substantial evidence” *against* location-specific spatiotopic TAE. For the retinotopic condition, this gave us the following: *M* = 29.96, *SE* = 5.87, *Bf* > 1 × 10^5^. This indicates “decisive evidence” *in favour of* location-specific retinotopic TAE.

To summarize the results of Experiment 2, we found that the location-specific component of TAE is retinotopic shortly after an eye movement: Tilt-adaptation is anchored to the retina (retinotopic) and not to locations in space (spatiotopic). However, one might argue that it takes some time for spatiotopic TAE to emerge after a saccade (i.e. it takes some time for the visual system to recover after an eye movement), and that the post-saccadic delay of 100 ms was too brief^4^,^40^. Therefore, in Experiment 3, we repeated the experiment, but presented the tester at a longer interval after the eye movement.

### Experiment 3

The aim of Experiment 3 was to investigate whether TAE is also retinotopic when the tester stimulus is presented at a longer (500 ms, in contrast to 100 ms in Exp. 2) interval after an eye movement. The experimental paradigm is described under *Methods*.

Trials were excluded based on the same criteria as in Experiment 2: The eyes deviated more than 2° from the expected location during the trial (8.4%); Saccade latencies were below 50 ms (6.1%) or above 500 ms (1.6%); Response times were below 50 ms (-) or above 1500 ms (1.5%). In total, 82.3% of all trials were included in the analysis.

The same repeated measures ANOVA as in Experiment 2 with TAE_acc_ as dependent variable revealed an effect of location, *F*(1,4) = 20.1, *p* = .0103, and trends toward an effect of condition, *F*(1,4) = 5.5, *p* = .0793, and a condition by location interaction, *F*(1,4) = 7.0, *p* = .0568 (see Figure 2b and Table 2). These effects were qualitatively identical to those found in Experiment 2. A similar analysis with TAE_rt_ as dependent variable revealed no effects, but yielded qualitatively similar results.

Crucially, we performed the same Bayesian analysis as in Exp. 2. For the spatiotopic condition, this gave us the following: *M* = 2.24, *SE* = 5.31, *Bf* = 0.19. Again, this indicates “substantial evidence” *against* location-specific spatiotopic TAE. For the retinotopic condition, this gave us the following: *M* = 22.61, *SE* = 4.01, *Bf* > 1 × 10^6^. Again, this indicates “decisive evidence” *in favour of* a location-specific retinotopic TAE.

To summarize the results of Experiment 3, we replicated the finding that the location-specific component of TAE is retinotopic. In addition, we excluded the alternative explanation that the lack of spatiotopic TAE in Experiment 2 was due to the short interval between saccade onset and tester presentation (i.e. a ‘retinotopic trace’-like phenomenon^4^,^6^).

## Discussion

The present results strongly suggest that the location-specific component of the tilt-adaptation aftereffect (TAE) is tied to a purely retinotopic frame of reference. Neither shortly (Exp. 2) nor at longer intervals (Exp. 3) following a saccadic eye movement did we find TAE at the originally adapted (spatiotopic) location. Crucially, our analysis suggests that this is not a null result due to a lack of statistical power: The combined data of experiments 2 and 3 is about 38 times (0.14^−1^ × 0.19^−1^, see *Results*) more likely to arise under a model without spatiotopic TAE than under a model with spatiotopic TAE, given a reasonable set of assumptions. Our results confirm a recent report of purely retinotopic TAE^33^, and are inconsistent with studies that have shown spatiotopic TAE across eye movements^23^,^25^,^41^. More generally, our results support the view that there is no transsaccadic integration (or ‘remapping’) of low-level visual features^3^,^8^,^9^.

Even in light of the present results, there is no obvious explanation for the fact that some studies have shown spatiotopic adaptation aftereffects across saccades^22^,^23^,^25^,^27^,^26^,^41^, whereas other studies, including the present one, have failed to find any such evidence^31^,^32^,^33^,^34^,^35^,^36^. One possibility is that spatiotopic aftereffects may sometimes emerge, but only when attention is focused on the adapter stimulus. This is indirectly supported by a recent functional magnetic resonance imaging study (fMRI) study, in which spatiotopic selectivity was reported in a range of visual brain areas^42^. Crucially, this spatiotopic selectivity was found only when attention was focused on the stimulus that elicited the activation, but not under conditions of passive viewing^43^,^44^. It might be that for some reason, such as subtle differences in the paradigm or instructions, participants sometimes pay attention to the adapter stimulus, while they ignore the adapter in other situations.

An alternative possibility, favoured by Knapen and colleagues^32^, is that generalised, non-location-specific adaptation aftereffects have occasionally been mistaken for location-specific, spatiotopic effects. This is supported by the observation that, in hindsight, none of the studies that have reported spatiotopic adaptation aftereffects have used a carefully controlled design. In particular, in these studies the spatiotopic and retinotopic selectivity has not been determined by comparing the adaptation effect to separate control locations, which have been matched in terms of eccentricity relative to both the first and second fixation^22^, and the direction of the saccade^23^.

While acknowledging that the issue is open to debate, we believe that the hypothesis that generalised adaptation aftereffects have been mistaken for location-specific, spatiotopic effects is the most parsimonious way to reconcile the divergent findings^33^. This also reduces the apparent gap between findings on adaptation aftereffects across saccades and the broader literature on visual stability and transsaccadic integration. More specifically, the consensus is that detailed, low-level information is mostly, if not entirely, lost across saccades^20^,^45^, whereas conceptual information is retained to some extent^18^, even though the spatial specificity of this form of transsaccadic integration is debatable^46^,^19^,^1^. In this view, spatiotopic TAE would be highly surprising.

In summary, we report that the location-specific component of the tilt-adaptation aftereffect (TAE) is purely retinotopic. After an eye movement, TAE is not found at the originally adapted location, but only at the location that retinotopically matches the adapted location. We have acknowledged that the issue is open to debate, but have suggested that previous reports of spatiotopic adaptation aftereffects have been due to an incorrect choice of control locations, which allowed generalised effects to be mistaken for location-specific, spatiotopic effects^22^,^23^,^25^,^41^. Finally, we have argued that the present results are consistent with the notion that there is little, if any, transsaccadic integration of low-level visual information^1^,^3^,^8^,^9^.

## Methods

### Experiment 1

8 observers, including one of the authors (SM), participated in the experiment. All participants reported normal or corrected visual acuity. Observers participated for course credit or monetary compensation. The experiment was conducted with approval of the Scientific and Ethical Review Board (VCWE) of the Faculty of Psychology and Education at the VU University Amsterdam, and was in accordance with the declaration of Helsinki. All participants provided informed consent prior to the experiment, and were debriefed afterwards.

Eye movements were recorded using an EyeLink 1000 (SR Research, Mississauga, Canada, ON), a video based eye tracker sampling at 1000 Hz. Stimuli were presented on a 22” CRT monitor, with a resolution of 1024 × 768 px and a refresh rate of 100 Hz.

**Figure 3:**
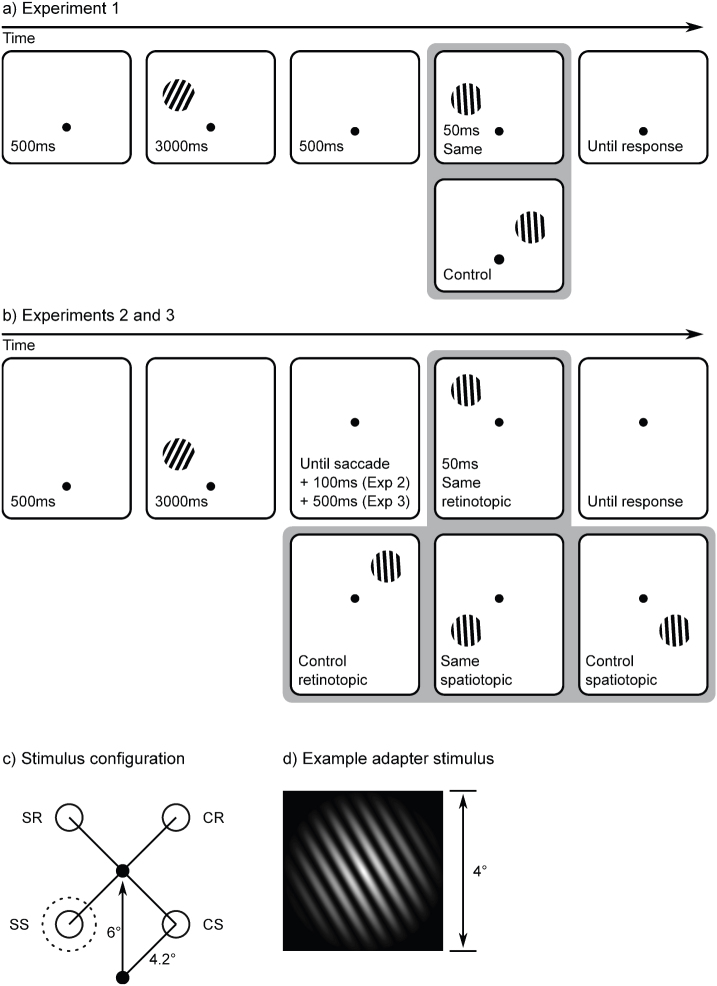
A schematic example of the experimental paradigm. (a) In Experiment 1, participants did not make any eye movements, and the tester could be presented either at the same location as the adapter, or at a control location. (b) In Experiments 2 and 3, participants made an eye movement in between the presentation of the tester and the adapter. The tester could be presented at either the same retinotopic location as the adapter, the same spatiotopic location as the adapter, or at one of two matched control locations. (Figure has been optimized for print, see text for stimulus details.) (c) A schematic of the stimulus configuration. The unfilled dotted circle indicates the adapter location. The unfilled solid circles indicate the four possible tester locations (SS: same spatiotopic; CS: control spatiotopic; SR: same retinotopic; CR: control retinotopic). The arrow indicates the displacement of the fixation dot. During the experiment, the stimulus arrangement was rotated and mirrored randomly. (d) An example adapter grating.

A schematic example trial is shown in Figure 3a. Before the start of each trial, a central white fixation cross was presented against a dark grey background. A drift correction procedure was triggered automatically as soon as a stable fixation was detected, except before the first trial of each block, in which a space bar press was required. Next, the trial proper started with the presentation of a central white fixation dot. After 500 ms, an adapter stimulus was presented for 3000 ms at a fully random location on an imaginary circle with a 4.2° radius, centred on the fixation dot. The adapter stimulus was a sinusoid luminance modulation with a spatial frequency of 2.5 cycles/°, maximum contrast, a linear envelope, a phase of 0, and a radius of 4° (see Figure 3d). The display was gamma corrected. The adapter was rotated clockwise or counterclockwise by 30° or 15° (angular) from a vertical orientation. 500 ms after the adapter was extinguished, a tester stimulus was shown for 50 ms. The tester was presented at the same location as the adapter stimulus, or at 6° distance from the adapter at the same eccentricity from the fixation dot. The tester was rotated clockwise or counterclockwise by 6°, 4°, or 2° (angular), and was otherwise identical to the adapter. Participants were instructed to report the orientation of the tester stimulus as quickly as possible by pressing the ‘z’ key on a counterclockwise rotation, and the slash-key on a clockwise rotation.

Tester orientation (2°, 4°, 6°), adapter orientation (15°, 30°), and tester location relative to adapter (same, different) were mixed within blocks. The experiment consisted of 384 trials, divided into 6 blocks, and was preceded by 24 practice trials.

### Experiment 2

The method was similar to that of Experiment 1, with the following exceptions. 8 new observers participated in the experiment. All were naïve as to the purpose of the study and none were trained psychophysical observers.

A schematic example trial is shown in Figure 3b. After the presentation of the adapter stimulus, the fixation dot was displaced 6° to a fully random location, with the constraint that the selected location was always 6.4° away from the display edge^cf.^ 40. Participants were instructed to make an eye movement towards the new location of the fixation dot. 100 ms after a saccadic eye movement had been detected, the tester stimulus was presented for 50 ms. There were four possible stimulus configurations (Figure 3c). In the *same spatiotopic* condition, the tester was presented at the same location as the adapter. In the *control spatiotopic* condition, the tester was presented at the location that mirrored the adapter location in the trajectory of the eye movement. In the *same retinotopic* condition, the tester was presented at the same retinal location as the adapter stimulus. In the *control retinotopic* condition, the tester was presented at the location that mirrored the retinal adapter location in the trajectory of the eye movement. The final fixation location was used as the initial fixation location for the next trial, so that the paradigm had the appearance of a random walk across the display.

The tester grating was always tilted 2° clockwise or counterclockwise. The adapter grating was always tilted 30° clockwise or counterclockwise. Condition (spatiotopic, retinotopic) and location (same, control) were randomly mixed within blocks. The experiment consisted of 384 trials, divided into 6 blocks, and was preceded by 24 practice trials.

### Experiment 3

The method was identical to that of Experiment 2, with the following exceptions. 5 new observers participated in the experiment. All were naïve as to the purpose of the study and none were trained psychophysical observers. The tester stimulus was presented 500 ms after the onset of a saccadic eye movement had been detected.

### Measure of tilt-adaptation aftereffect (TAE)

In a typical tilt-adaptation experiment, the orientation of the tester stimulus is perceived as being tilted slightly away (relative to its actual orientation) from the orientation of the adapter stimulus. In other words, the orientation of the tester will seem more pronounced when it is preceded by an adapter that is oriented in the opposite direction (incongruent trials; e.g., a 2° tester and a −30° adapter), compared to when it is preceded by an adapter oriented in the same direction (congruent trials; e.g., a −4° tester and −15° orientation). The more pronounced the orientation of the tester appears, the faster and more accurate participants will respond. Therefore, in the current paradigm TAE can be measured as a reverse congruency effect.

For accuracy, TAE was determined as follows (high values reflect strong TAE): 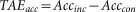


Here *con* are congruent trials, and *inc* are incongruent trials. For response times, TAE was determined as follows (high values reflect strong TAE): 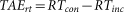


### Measure of location-specific tilt-adaptation aftereffect (LSTAE)

TAE is a largely localised effect, but also has a weaker non-location-specific component. In the present study, in particular in Exp. 1, it is therefore crucial that we have a sensitive measure of the location-specific TAE (LSTAE).

For response times, LSTAE was determined as follows: 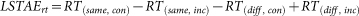


Here *RT* is the mean correct response time, *same* is the same location condition, *diff* is the different location condition, *con* are congruent trials, and *inc* are incongruent trials (for a description of the conditions, see *Methods → Experiment 1*). For accuracy, LSTAE was determined as follows: 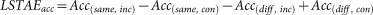


Here, *Acc* is the proportion of correct trials.

## Author Contributions

S.M. and J.T. wrote the manuscript. S.M. programmed the experiment, and collected and analysed the data.

## Figures and Tables

**Table 1:** Individual accuracy scores (%) for Experiment 2

Participant	Condition							
	Same ret.		Control ret.		Same spa.		Control spa.	
	Inc.	Con.	Inc.	Con.	Inc.	Con.	Inc.	Con.
1	89.1	42.5	92.5	55.0	82.2	70.0	81.0	59.0
2	89.4	50.0	85.1	82.2	84.4	78.7	80.0	85.1
3	92.3	29.7	95.2	85.0	75.0	78.6	90.0	97.1
4	76.2	42.9	66.7	51.2	85.7	70.0	66.7	68.3
5	92.5	56.8	83.3	76.3	76.9	78.0	82.8	76.3
6	95.7	57.4	91.3	83.7	93.3	84.1	89.1	67.4
7	95.5	42.9	81.4	81.0	83.3	81.8	88.6	85.4
8	93.5	70.6	69.0	58.3	71.4	62.1	76.9	80.8
**Mean**	**90.5**	**49.1**	**83.1**	**71.6**	**81.5**	**75.4**	**81.9**	**77.4**

Inc.: incongruent; Con.: congruent; ret.: retinotopic; spa.: spatiotopic.

**Table 2:** Individual accuracy scores (%) for Experiment 3

Participant	Condition							
	Same ret.		Control ret.		Same spa.		Control spa.	
	Inc.	Con.	Inc.	Con.	Inc.	Con.	Inc.	Con.
1	86.4	57.1	85.3	74.4	92.9	73.7	92.1	83.0
2	70.5	39.5	72.1	53.7	62.5	60.5	56.8	68.9
3	100.0	68.2	93.3	97.7	95.0	81.8	95.5	88.4
4	74.4	48.8	80.5	81.0	71.7	77.5	82.1	72.3
5	77.8	63.3	62.1	67.7	55.2	58.6	63.0	62.9
**Mean**	**81.8**	**55.4**	**78.7**	**74.9**	**75.5**	**70.4**	**77.9**	**75.1**

Inc.: incongruent; Con.: congruent; ret.: retinotopic; spa.: spatiotopic.
